# Synovial fluid *α*-defensin might be false positive in early stages after major total arthroplasty revision surgery

**DOI:** 10.5194/jbji-11-1-2026

**Published:** 2026-01-07

**Authors:** Sankalp Mrutyunjaya, Wade A. Banta, Joseph R. B. Espiritu, Derek F. Amanatullah

**Affiliations:** 1 Department of Orthopaedic Surgery, Stanford Medicine, Redwood City, CA, USA; 2 School of Medicine, Stanford University, Palo Alto, CA, USA

## Abstract

Generalizability is critical when evaluating the performance of a diagnostic test to ensure variations in the patient population are represented. We report a case of a patient receiving multiple false positive results from the synovial α -defensin test observed over close to a 3-year period following revision total knee arthroplasty.

## 1 Introduction

Generalizability is the ability to infer results from one set of circumstances from a clinical trial and apply them to other settings (Davis, 1994). This concept is critical when evaluating the performance of a diagnostic test. Clinical trials used to evaluate diagnostic tests typically involve small subsets of patients which may not encompass the diversity represented by the target clinical demographic (Davis, 1994). The effect of generalizability is usually applied to variations in the patient population.

Generalizability can also include healthcare provider acceptance and the proper application of a diagnostic test (Uy, 2022). Overgeneralization may skew pre-test probability having an impact on the final diagnosis. Hence, a mismatch between the provider's perceived pre-test probability and the revised probability after receiving the test results can potentially extend the cost and duration of care. Therefore, it is important that clinicians using diagnostic tests consider pre-test probability while maintaining high accuracy in diagnosis to optimize the use of healthcare resources to treat patients (Uy, 2022).

Here we look at a case where pre-test probability can affect the use of a diagnostic test, synovial α -defensin (S α D), designed for detection for periprosthetic joint infections (PJIs) (Rycyk-Bojarzyńska et al., 2024; Stone et al., 2019). The diagnosis of periprosthetic joint infection typically involves monitoring serum erythrocyte sedimentation rate (ESR) and C-reactive protein (CRP) levels, along with intra-articular recruitment of polymorphonuclear cells by bacteria (Stone et al., 2019). S α D is an immunoassay that measures the intra-articular concentration of the α -defensin peptide released during the presence of a pathogen (Rycyk-Bojarzyńskaet al., 2024). In 2018, S α D was added to the Musculoskeletal Infection Society (MSIS) criteria; our case report shows a false positive S α D result and serves as an example of how overgeneralization of any highly specific tests has the potential to influence patient care (Kim and Cho, 2021).

## 2 Case history

A 55-year-old male presented with a history of type II diabetes mellitus, obesity (body mass index: 35.3 kg m^−2^), hypertension, and multiple orthopedic procedures – including right shoulder arthroplasty, left Achilles tendon debridement, right multi-ligamentous knee reconstruction, patellar tendon lengthening with patellectomy, and an eventual total knee arthroplasty (TKA) – in December 2016. The patient was involved in a motor vehicle accident in November 2020, resulting in progressive pain, swelling, instability, and extensor lag ( > 30°) of his right TKA. He underwent revision TKA for global instability with extensor mechanism reconstruction in March 2021.

**Figure 1:**
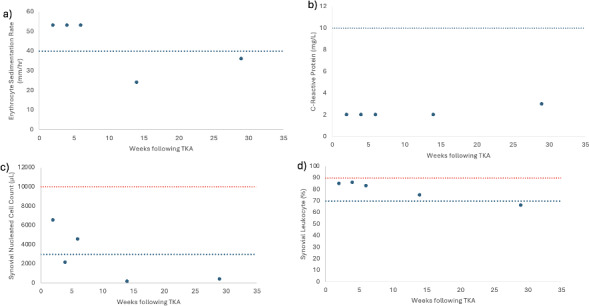
**(a)** ESR levels, **(b)** CRP levels, **(c)** synovial leukocyte count, and **(d)** percentage of synovial leukocytes reported based on the postoperative week tested. The dotted blue line indicates 2018 MSIS thresholds for each laboratory value (Prince et al., 2020). The dotted red line in panels **(c)** and **(d)** indicates the threshold for PJI within 6 weeks (Prince et al., 2020; Rycyk-Bojarzyńska et al., 2024).

Given the patient's numerous knee incisions from his prior multi-ligamentous knee reconstruction, the distal aspect of his midline incision over the tibial tubercle exhibited poor incisional healing 2 weeks after revision surgery, with a skin bridge of < 7 cm from a more lateral incision. This was treated with dry dressings, and oral antibiotics were not started. At this time, serum markers and a synovial aspiration were drawn to evaluate for an infection (Fig. 1, Table 1). All tissue and synovial aspirations are routinely held for 14 d to evaluate for fastidious organisms, like *Cutibacterium acnes*, and, in patients with risk factors for immunosuppression, any fungal cultures are held for 30 d.

The patient was readmitted 4 weeks after surgery with cellulitis at the right shin and new blistering at the distal aspect of the incision. A synovial aspiration revealed negative aerobic and anaerobic cultures, improving aspirate with respect to synovial nucleated cell count, and a second positive S α D test (Fig. 1c and d, Table 1). He was subsequently diagnosed with cellulitis in the distal tibia, together with congestive heart and liver failure (e.g., hepatosplenomegaly and hepatic steatosis). Infectious disease consultation recommended monitoring the patient off antibiotics and repeat testing at 6 weeks (Fig. 1, Table 1). Given the early interval, acute thresholds for PJI were applied (Bingham et al., 2014).

For 3 months after surgery, the patient continued with bilateral pitting edema and an elevated jugular venous pressure, necessitating urgent diuresis in the emergency department. Repeat serologies and synovial fluid aspiration then showed improvement and a negative S α D test in correlation with wound healing (Fig. 1, Table 1). At this time, the patient no longer met the MSIS infection criteria. Testing for CRP and ESR was performed 143 weeks after surgery, and the patient remained infection free (ESR: 16 mm h^−1^; CRP: 2.9 mg L^−1^; Table 1).

**Table 1:** Synovial CRP and non-quantitative infection markers and 2018 MSIS score at each postoperative week accounting for the acute infection within 6 weeks.

	2 weeks	4 weeks	6 weeks	14 weeks	29 weeks	143 weeks
S α D	Positive	Positive	Positive	Negative	Negative	Negative
Synovial CRP	1.9 mg L^−1^	1.1 mg L^−1^	0.8 mg L^−1^	≤0.4 mg L^−1^	≤0.4 mg L^−1^	≤0.4 mg L^−1^
Culture	Negative	Negative	Negative	Negative	Negative	Negative
MSIS score	1^*^	1^*^	1^*^	2	0	0
Synovial nucleated cell count	6533	2139	4556	178	416	
Synovial leukocyte (%)	85	86	83	75	66	

^*^ Indicates the application of an elevated threshold for PJI within 6 weeks (Kumar et al., 2023).

## 3 Discussion

TKA is one of the most common surgeries performed in the United States (Weinstein et al., 2023). PJI is one of the most common reasons for revision arthroplasty (Heckmann et al., 2023). PJIs occur in about 2.4 % of total joint replacements and represent a burden to patients and the healthcare system (Suen et al., 2018). PJIs are diagnosed in accordance with MSIS criteria which look at serum and synovial biomarkers (Heckmann et al., 2023). However, S α D testing looks specifically for the α -defensin peptide present in the synovial fluid (Suen et al., 2018). In the patient case presented above, we observe S α D showing a false positive result for PJI.

In observing the patient's laboratory results, the S α D test performed prior to postoperative week 6 consistently showed positive results. Although this result would appear to be indicative of a PJI, other laboratory results for CRP and synovial leukocyte count show less consistency with the S α D test, making the diagnosis less clear. CRP trends throughout the postoperative period show no abnormal values, while synovial leukocyte cell count shows initial fluctuation through weeks 2 and 6. These inconsistencies follow when observing calculated MSIS scores in which weeks 2 and 6 in the postoperative period would be indicative of PJI, while week 4 would not be indicative of PJI. PJI in an acute setting ( < 90 d post-operation) would show a synovial leukocyte cell count greater than 10 000 and a synovial leukocyte percentage greater than 90 % (Kim and Cho, 2021). However, weeks 2 through 6, which are within 90 d of the postoperative period, consistently show values below this threshold for acute PJI (Fig. 1). Ultimately, all laboratory measurements trend below the abnormal threshold by week 29 of the postoperative period. With the patient's aspirated synovial fluid also being negative for aerobic and anaerobic bacterial cultures in all instances, it is more likely that the patient never had PJI and that the presence of α -defensin peptide can be potentially attributed to other causes unrelated to a PJI that stimulated the innate immune system.

The S α D results in this case are likely false positives. The effects of both the surgical wound (acute postsurgical insult of a TKA and the presence of an allograft extensor mechanism reconstruction) and the circumstances of the patient (liver failure and congestive heart failure) on S α D have not been formally evaluated (Kumar et al., 2023). Hence, S α D, in the context of evaluating PJI, may not be used exclusively as an MSIS minor criterion to diagnose PJI because of multiple confounding factors that may lead to variation in the α -defensin levels. This variation in α -defensin levels only strengthens the mismatch between the provider's pre-test probability and revised probability after S α D due to the inconsistency between a positive S α D and negative culture result and downward trend in the levels of multiple biomarkers. Although the qualitative synovial α -defensin test (e.g., positive/negative) has a sensitivity of ≥ 97 %, a specificity of ≥ 95 %, a positive predictive value of ≥ 88 %, a negative predictive value ≥ 99 %, and an area under the curve of ≥ 0.99, we can see that despite the high sensitivity and specificity, S α D may add little to or even limit the traditional serological and synovial data work-up used to diagnose a PJI (Bingham et al., 2014). As such, this highlights the importance of critically evaluating different tests involved in the potential diagnosis of a failed arthroplasty. Other studies have noted issues of false positive results with S α D results due to non-infectious inflammatory disease, such as acute gout, or the inability to reliably distinguish between septic and inflammatory arthritis (Table 2) (Partridge et al., 2017; Plate et al., 2020; Cooper et al., 2021). With the clinical utility of S α D in question, using α -defensin to diagnose PJIs indiscriminately can easily increase the burden on the patient due to a lack of generalizability and falsely confirming a physician's pretest probability.

**Table 2:** Other studies reporting on false positive S α D results.

Authors	Year	Concern for false positive S α D
Partridge et al. (2017)	2017	Gout after total knee arthroplasty
Plate et al. (2018)	2018	Inflammatory arthritis
Cooper et al. (2021)	2021	Native knee joint infection

## Data Availability

The datasets generated and analyzed during this case report are not publicly available because they were collected under institutional review board approval for a single, potentially identifiable patient. Public release could compromise patient anonymity. Accordingly, the datasets are available from the corresponding author upon reasonable request and following implementation of appropriate safeguards to protect patient confidentiality.
